# Variable window binding for mutually exclusive alternative splicing

**DOI:** 10.1186/gb-2006-7-1-r2

**Published:** 2006-01-13

**Authors:** Dimitris Anastassiou, Hairuo Liu, Vinay Varadan

**Affiliations:** 1 Center for Computational Biology and Bioinformatics, and Department of Electrical Engineering, Columbia University, New York, NY 07670, USA

## Abstract

**Background:**

Genes of advanced organisms undergo alternative splicing, which can be mutually exclusive, in the sense that only one exon is included in the mature mRNA out of a cluster of alternative choices, often arranged in a tandem array. In many cases, however, the details of the underlying biologic mechanisms are unknown.

**Results:**

We describe 'variable window binding' - a mechanism used for mutually exclusive alternative splicing by which a segment ('window') of a conserved nucleotide 'anchor' sequence upstream of the exon 6 cluster in the pre-mRNA of the fruitfly *Dscam *gene binds to one of the introns, thereby activating selection of the exon directly downstream from the binding site. This mechanism is supported by the fact that the anchor sequence can be inferred solely from a comparison of the intron sequences using a genetic algorithm. Because the window location varies for each exon choice, regulation can be achieved by obstructing part of that sequence. We also describe a related mechanism based on competing pre-mRNA stem-loop structures that could explain the mutually exclusive choice of exon 17 of the *Dscam *gene.

**Conclusion:**

On the basis of comparative sequence analysis, we propose efficient biologic mechanisms of alternative splicing of the *Drosophila Dscam *gene that rely on the inherent structure of the pre-mRNA. Related mechanisms employing 'locus control regions' could be involved on other occasions of mutually exclusive choices of exons or genes.

## Abstract 2

A mechanism called Variable Window Binding for mutually exclusive alternative splicing is described for exon 6 of the *Drosophila Dscam* gene, which represents an extreme case of alternative splicing.

## Background

Alternative splicing and its regulation are topics of increased interest because of the evidence that most genes of advanced organisms adopt this mechanism in response to developmental and cellular contexts to express molecularly distinct transcripts [1,2]. In some cases, splicing is mutually exclusive, such that only one exon is chosen out of a cluster of alternative exons arranged in a tandem array [3]. Given that alternative splicing malfunction can lead to serious human genetic diseases [4], efforts to unravel the underlying biologic mechanisms could lead to valuable therapeutic strategies to suppress these defects.

**Figure 1:**
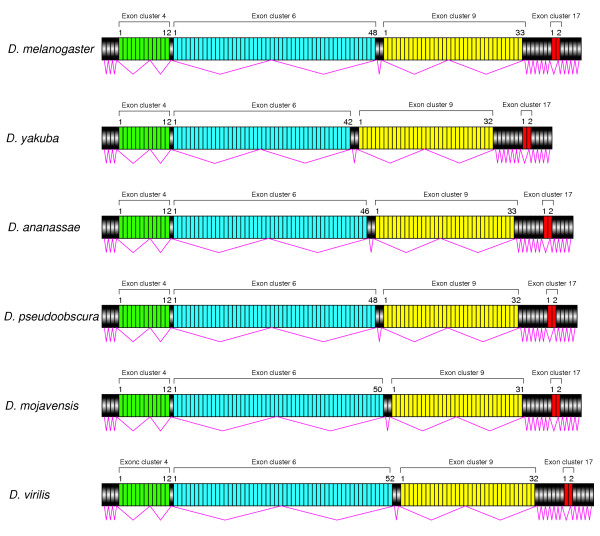
The structure of the *Dscam *gene in six *Drosophila *spp. The mature mRNA of each gene contains 22 exons, four of which (exon 4, exon 6, exon 9 and exon 17, shown in green, blue, yellow and red, respectively) are chosen from a cluster of alternative exons. The remaining 18 exons, shown in metallic gray, are always selected. Introns are not shown. Red lines indicate an example choice of exons.

An extreme case of mutually exclusive alternative splicing occurs in the *Drosophila *Down syndrome cell adhesion molecule (Dscam) gene [5], which encodes an axon guidance receptor of the immunoglobulin superfamily with numerous possible isoforms, which probably play key roles in the nervous system of the fly [6-8]. Recent results indicate that Dscam also functions in the immune system [9,10]. In insect species, the organization of the *Dscam *gene is very similar but not identical [11]. Here, we have selected six fully sequenced *Drosophila *spp. (Figure 1): *D. melanogaster*, *D. yakuba*, *D. ananassae*, *D. pseudoobscura*, *D. mojavensis*, and *D. virilis*. In each of these six species, the *Dscam *gene contains four clusters of alternative ('variable') exons. These are the clusters containing exon 4, exon 6, exon 9 and exon 17, selected out of 12, 48, 33, and 2 alternative exons, respectively, in *D. melanogaster*.

We use the following notation. Starting, for example, from the variable exon 6 that is closest to the constant exon 5, we refer to these exons as exon 6.1, exon 6.2, exon 6.3, and so on. Similarly, we refer to the partial introns between two consecutive exons using the numbers of these exons. For example, the partial intron between exons 6.32 and 6.33 is referred to as intron 6.32-33. Similarly, we refer to the partial intron between constant exon 5 and variable exon 6.1 as intron 5-6.1.

Using comparative sequence analysis, we discovered the basis of a novel biologic mechanism, to which we refer as 'variable window binding' (VWB), that could account for the mutually exclusive selection of one exon from among the members of cluster 6, as well as a related mechanism to explain the mutually exclusive selection of one exon out of the two members of cluster 17.

## Results

### Discovery of anchor sequence

Because the *Dscam *gene of four out of the six *Drosophila *spp. had not previously been annotated [11], we first generated the missing annotations for all exons of cluster 6 using the existing annotations as benchmarks and ensuring that exons are located in open reading frames. The resulting annotated sequences for *D. yakuba*, *D. ananassae*, *D. mojavensis *and *D. pseudoobscura *have been deposited in GenBank under accession numbers DQ317106, DQ317107, DQ317108 and DQ317109, respectively. These can be accessed in addition to the previously available annotated sequences for *D. melanogaster *(accession number AF260530) and *D. virilis *(accession number AY686597).

We then identified the homologous relationship of all cluster 6 variable exons in all six species, resulting in a 'multiple exon alignment' (Table 1). This relationship is orthologous among similar exons of different species, and paralogous for exons in the same species [11], because exons are assumed to have been created by reiterative duplication events [3]. We derived the multiple exon alignment after first generating a phylogenetic tree (the generated tree is provided in Additional data file 1) with leaves corresponding to conceptually translated amino acid sequences for all exons [11]. Because the six species are closely related, almost all of the clusters of neighboring leaves of the tree correspond to orthologous exons of different species, which can thus be conveniently aligned.

Based on the multiple exon alignment, we then created a 'multiple intron alignment' by aligning a set of introns if each of them is located directly upstream of a member of a particular set of orthologous exons. We then searched for potential *cis*-regulatory motifs by identifying the largest subsequences that were conserved in all six species in each set of orthologous introns. We found that intron 5-6.1 contains a large subsequence of length 42, henceforth referred to as the 'anchor sequence', which is precisely conserved among all six *Drosophila *spp.:

AAAUUGAAAACUGCCUGAAUGUUGGGAUAGGGUACUCGACAA

### Reverse complementary motif detection

Remarkably, we observed that many of the conserved motifs that we found in the sets of orthologous introns were the reverse complement of part of the anchor sequence. This observation led us to the hypothesis, which we confirmed computationally, that a binding site for a subsegment, or 'window', of the anchor sequence is located upstream of nearly all exons of cluster 6. There were very few exceptions to this rule, such as the intron upstream of exon 6.11 of *D. melanogaster*, which is probably non-functional because its expression has not been detected [12,13].

Because the window of the anchor sequence binding on the intronic elements is different for each exon choice, it has been difficult for these intronic binding sites to be detected as conserved motifs. Any isolated observations that part of the intron 5-6.1 is complementary to a particular seemingly nonconserved region of another intron can be dismissed as being due to chance, but not if this phenomenon happens for nearly all introns of all *Drosophila *spp. By using the systematic methodology described above, we isolated the individual sets of orthologous introns, many of which conserve the location of the anchor sequence window, and so these intronic elements were readily detected as conserved motifs.

### Variable window binding

**Figure 2:**
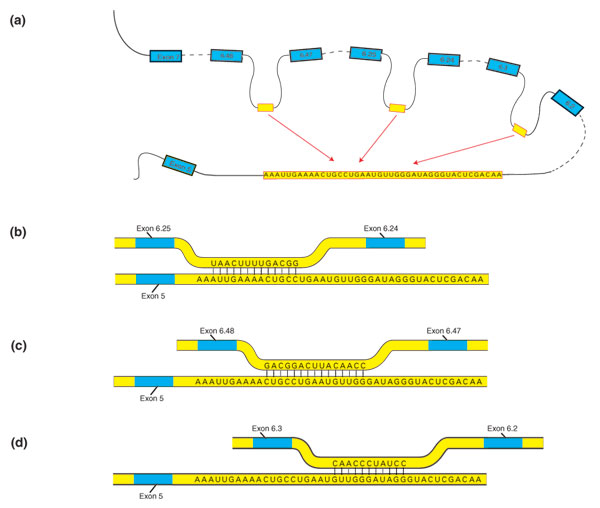
Illustration of variable window binding using sequence data from *Drosophila melanogaster*. The anchor sequence (conserved in all six species) is located downstream from exon 5 and is shown as the 'lower' strand, whereas its binding site is located upstream of the exon to be selected and is shown as the 'upper' strand. There are multiple alternative mutually exclusive pre-mRNA local secondary structures, three of which are shown. The figure is not drawn to scale. **(a) **Competition among multiple regions for pairing with the anchor sequence provides mutual exclusivity. **(b) **The binding site when exon 6.25 is selected to be connected with exon 5 corresponds to a window close to one end of the anchor sequence. **(c) **The binding site when exon 6.48 is selected to be connected with exon 5 corresponds to a window in the middle of the anchor sequence. **(d) **The binding site when exon 6.3 is selected to be connected with exon 5 corresponds to a window close to the other end of the anchor sequence.

The occurrence of regions within exon 6 cluster introns with striking complementarity to the anchor sequence downstream from exon 5 naturally suggests a biologic mechanism (Figure 2) that explains the mutual exclusivity of alternative splicing of exon 6. We refer to this mechanism as 'variable window binding' (VWB). A loop is created by base pairing between a short segment or 'window' of the anchor sequence with a complementary motif within an exon 6 intron, thereby allowing the spliceosome to connect constant exon 5 with the variable exon 6 now in proximity to the anchor sequence.

**Figure 3:**
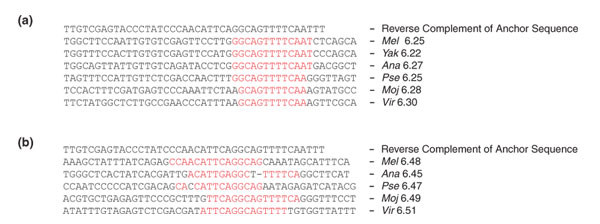
Multiple alignment of the binding sites of orthologous introns showing conservation and drift. **(a) **The sequence 5'-GGCAGUUUUCAA-3' upstream of exon 6.25 of *Drosophila melanogaster *is perfectly conserved in the orthologous exons (Table 1) across all species. **(b) **The binding sequence upstream of exon 6.48 of *D. melanogaster *drifts into different binding sequences in the orthologous exons of other species. Areas indicated in red are the predicted binding sites.

Binding sites correspond to different but usually overlapping 'windows' from the anchor sequence. This mechanism is compatible with the fact that the choice of the exon is 'stochastic yet biased', in accordance with observed expression data [12] in single cells. The exon choice may be influenced by the existence of various splicing factors in a given cell type. For example, splicing inhibitors could bind at a location close to, and obstructing, a given binding site region of the anchor sequence. The exon choice may also depend on the precise location of the window in the anchor sequence that binds on the intron. For example, if the 'first half' of the anchor sequence is obstructed from binding by a macromolecule or macromolecular assembly (or knocked out in a genetic experiment), then (Figure 2a) exons 6.25 and 6.48 of *D. melanogaster *cannot be chosen, but exon 6.3, as well as some other exons, can be selected. We refer to this model as 'VWB regulation' of alternative splicing. There are indications that VWB provides at least part of the regulatory mechanisms in exon cluster 6, particularly because the location of the windows across some orthologous introns is conserved to different degrees. For example, the sequence 5'-GGCAGUUUUCAA-3' upstream of exon 6.25 (Figure 2b) is perfectly conserved in all six species (Figure 3a), whereas in some other orthologous introns we observe 'drifting' of the window location. For example, the sequence 5'-CCAACAUUCAGGCAG-3' upstream of exon 6.48 of *D. melanogaster *(Figure 2c) drifts into 5'-AUUCAGGCAGUUUU-3' in the orthologous exon 6.51 of *D. virilis*, and further into 5'-UUCAGGCAGUUUUCA-3' in the orthologous exon 6.49 of *D. mojavensis*, thus relocating into a different window of the anchor sequence (Figure 3b). This fact suggests that the exons corresponding to the former introns (of conserved window location) are at least partly regulated by VWB, because the location of their window is vital, indicating that only the corresponding segment of the anchor sequence is available for binding, while the remaining part is probably obstructed by a regulatory molecule. On the other hand, the exons corresponding to the latter introns (of drifting window location) are regulated by other types of splicing factors, or are not regulated at all.

**Figure 4:**
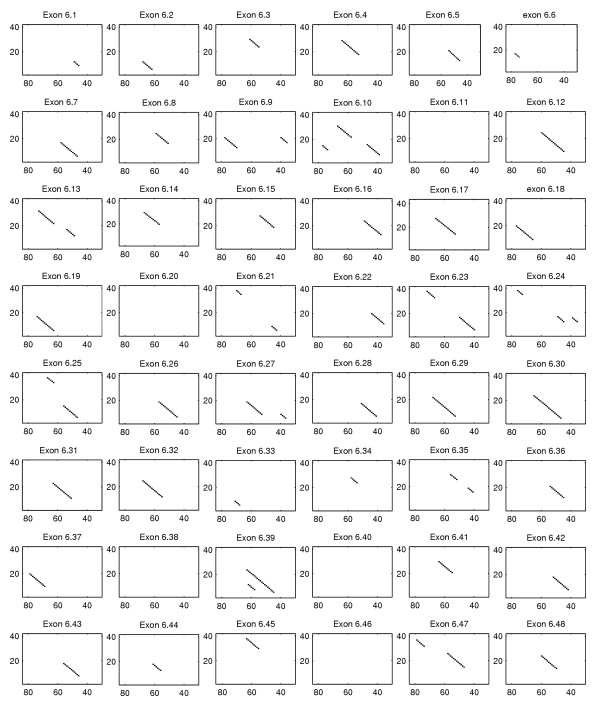
Dot plots illustrating the binding sites upstream of each alternative exon 6 in *Drosophila melanogaster*. The horizontal axis shows the distance in nucleotides from the exon to be chosen. The vertical axis indicates the nucleotides of the anchor sequence. The dot plots were created using the '8 out of 11' method and were further processed to filter out any dots that are not part of a continuous line of length at least 4. Line lengths should be seen as approximate indicators and not as precise measures of binding strength.

The existence of binding sites in nearly all exons is illustrated in dot plots (Figure 4). The precise predicted interactions of the anchor sequence with the sequences extending up to 120 nucleotides upstream of each exon is shown in Additional data file 2, created by performing Smith-Waterman alignment with the reverse complement of the anchor sequence. For easier interpretation of the figure, matches were highlighted in red color, and consecutive matches of length greater than five were highlighted in bold red color. The resulting alignment scores are shown in each case and can be seen as approximate indicators of binding strength. We found that the score of the alignment between the reverse complement of the anchor sequence and a random sequence of length 120 has mean 7.0 and standard deviation 1.3, implying that, with high confidence, the 41 out of 48 predicted binding sites with scores higher than 10 are not due to chance. Binding sites have an average distance of 60-70 nucleotides from the selected exon, and a few appear to extend in the exon upstream of the exon to be selected (indicated by gray shading). Among the seven exons with relatively low alignment score, we believe that binding sites are missing from at least two of them, namely 6.1 and 6.11, for the following reasons.

We found that the alignment scores for exons 6.1 in all six species were consistently low (10, 6, 8, 9, 9 and 9 for *D. melanogaster*, *D. yakuba*, *D. ananassae*, *D. psedoobscura*, *D. mojavensis *and *D. virilis*, respectively). Therefore, the choice of the first exon may exceptionally not require interaction with the anchor sequence.

Exon 6.11 of *D. melanogaster *appears to be nonfunctional, because its expression has never been observed [5,8,10,12,13] and because it is isolated in the phylogenetic tree (Additional data file 1). Such exons are labeled by an asterisk in Table 1, and they also include exons 6.10, 6.36 and 6.42 of *D. yakuba*. (Interestingly, exons 6.11 of *D. melanogaster *and 6.10 of *D. yakuba *appear closely connected to each other if the phylogenetic tree - not shown - is made directly from DNA and not from conceptually translated sequences, suggesting an evolutionary scenario leading to both of them becoming non-functional.) For all three such exons of *D. yakuba*, we observed that their upstream introns did not contain binding sites for the anchor sequence, and their size had been significantly reduced (35, 27 and 47 for 6.10, 6.36 and 6.42, respectively).

These observations are consistent with the hypothesis that the existence of a complementary binding site is a requirement for exon selection (except for exon 6.1), and therefore partial obstruction of the anchor sequence will influence exon selection. Interestingly, a few of the introns appear to have two binding sites (Figure 4), which may be a mechanism overcoming VWB regulatory inhibition of the selection of those exons.

### A computational strategy to reconstruct the anchor sequence from intron sequences

**Figure 5:**
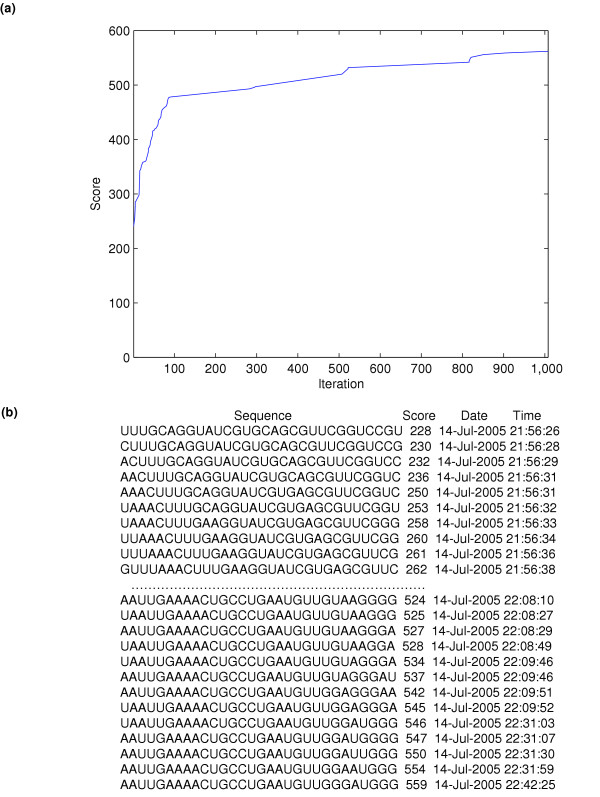
Output of the genetic algorithm showing convergence to anchor sequence. Starting from a completely random sequence, the genetic algorithm is able to converge to a sequence that is a substring of the full anchor sequence. **(a) **The fitness progression of one of the runs of the genetic algorithm. The genetic algorithm searches in the space of all possible sequences to converge to a high fitness sequence that matches the anchor sequence. **(b) **The evolution of the estimated anchor sequence at each iteration of the genetic algorithm. The output converges to a substring of the actual anchor sequence, namely AAAUUGAAAACUGCCUGAAUGUUGGGAUAGGGUACUCGACAA.

Given the sequences of the introns of cluster 6, can we reconstruct the anchor sequence, assuming no previous knowledge about it? For example, if we had not revealed the anchor sequence, could someone derive it using only knowledge of, say, introns 6.1-2, 6.2-3 ... 6.47-48 of *D. melanogaster*? The answer is 'yes'. Using a 'genetic algorithm' (software implemented in MATLAB is provided in Additional data file 3), one can reconstruct the anchor sequence by maximizing a score ('fitness function') assigned to nucleotide sequences of a particular length. The score is defined as the sum of the scores of a local (Smith-Waterman) alignment between the complementary sequence and the known introns. If an initial random 'seed' sequence undergoes random 'mutations' (substitutions, insertions, and deletions) and 'survives' only if these mutations improve the score (Figure 5a), then it becomes gradually 'mutated' into the reverse complement of the anchor sequence (Figure 5b). The sequence converges to the reverse complement of the same anchor sequence in each of the six cases where the intron inputs are only taken from one species. This technique can be used to reconstruct the anchor sequences from other potential cases of VWB-based mutually exclusive selection of exons, providing the most convincing argument next to genetic experimentation that these binding sites are real.

### Potential competing stem structures for the selection of exon 17

The fact that pre-mRNA alternative secondary structures, the choice of which can be stochastic and influenced by splicing factors, can determine alternative splicing selections is increasingly appreciated [14], and VWB-based alternative splicing is such a case. A similar mechanism involving internal base pairing interactions between distal segments of the pre-mRNA could also account for the binary choice between exon 17.1 and 17.2 of the *Drosophila Dscam *gene (Figure 1).

**Figure 6:**
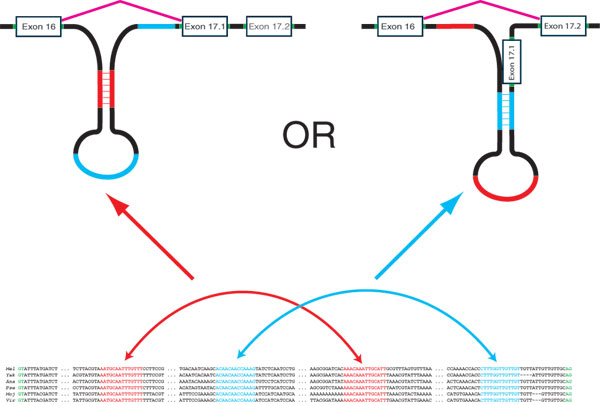
Illustration of a potential mechanism for the selection of exon 17. When the stem from the blue sequences is implemented, it potentially obstructs the splicing branch point of intron 16-17.1 inhibiting the selection of exon 17.1 and resulting in the selection of exon 17.2. If the competing stem from the red sequences is implemented, then, under the additional assumption that a pseudoknot involving both interactions is not implemented, exon 16 is allowed to be spliced directly into exon 17.1.

We have identified two pairs of complementary conserved motifs on intron 16-17.1 (AAATGCAATTTGTTT with AAACAAATTGCATT, and ACAACAACCAAAG with CTTTGGTTGTTGT). As shown in Figure 6, the choice of a particular binding pair could be part of the mechanism determining whether exon 17.1 or exon 17.2 is selected. If the latter stem (via binding of the blue sequences in Figure 6) is implemented, then it potentially obstructs the splicing branch point of intron 16-17.1, thereby inhibiting the selection of exon 17.1 and resulting in the selection of exon 17.2. On the other hand, if the stem is implemented via binding of the red sequences, then exon 16 could be spliced directly to exon 17.1. The presence of these two competing stem structures could constitute a novel mechanism involved in the mutually exclusive choice of only two exons. However, this is not as clear as with the exon 6 cluster, in which there was a unique anchor sequence, because it is possible for both of them to occur simultaneously through the formation of a pseudoknot. If this is indeed the underlying mechanism, then splicing regulation of exon 17 could be achieved by splicing factors, such as microRNAs or proteins, that bind on one of these sites (for instance, red or blue), resulting in the selection of the secondary structure involving the unobstructed stem.

## Discussion

We have performed extensive comparative sequence analysis looking for conserved motifs throughout the *Dscam *pre-mRNA among six *Drosophila *spp. This search revealed several perfectly conserved motifs, some of which appear as complementary pairs, which may lead to stem structures. The functionality of several of the conserved motifs and potential stem structures is not obvious, but in the case of exons 6 and 17 they suggested elegant biologic mechanisms of competing secondary structures that could help to explain the mutually exclusive choice of these exons. The roles of some stem structures in regulating alternative splicing were also recently shown [15] to be that they bring in proximity the two flanking sequences.

For example, we found a large (66 nucleotides) genomic region of unknown functionality perfectly conserved across all six *Drosophila *spp. at the beginning of constant exon 18:

GACCGGATTAAGCGAG|GTACAGTCATAAGTAAGCATCTAAATTTCCAATGCAACATTTATTAATGC

The vertical line indicates the border between the end of exon 18 and the beginning of intron 18-19. Because both exons 18 and 19 are constant, we speculate that this, previously not noted, conserved area could be involved in the formation of the general initial secondary structure of the pre-mRNA. We note that two smaller conserved motifs (TAAATGTTG and ATTGGAAATT), also on intron 18-19, are both complementary to segments of this large motif.

As another example, in intron 17.2-18 there are two pairs of complementary conserved motifs (AAAATATACCAAC with GTTGGTATATTTT, and AAGATGCTTTT with AAAAGCATCTT), which, as was the case with intron 16-17.1, are arranged in a manner suggesting either two mutually exclusive stems or a pseudoknot.

As for exon clusters 4 and 9, they appear to be more regulated compared with exon 6 [12,16], in the sense that most of their exons appear at varying degrees of expression at various tissues and developmental stages. We found that intron 4.12-5 contains several perfectly conserved large motifs, such as CCCTTCATGTAGTTGAA and CAAAAATGCTAATAA, with the latter one offering a potential binding site for another, smaller, conserved, complementary motif, GCATTTTTG, also in intron 4.12-5.

As in exon 6, many of the corresponding sets of orthologous introns within clusters 4 and 9 contain conserved motifs; however, these do not appear to be complementary to any anchor sequence. We propose that some of these motifs serve as binding sites of an intervening activating protein or complex, which is itself activated by first binding to an 'anchor sequence'. Under this hypothesis, as in the VWB-controlled case of exon 6, mutual exclusivity can be partly explained by the pre-mRNA loop uniquely connecting an anchor sequence with these binding sites.

The arrangement of a tandem array of mutually exclusive exons in the middle of a pre-mRNA molecule is strikingly similar to the arrangement of a tandem array of genes expressed in a mutually exclusive manner, suggesting the possibility that mutual exclusivity is implemented using similar mechanisms. Specifically, we propose that an 'activating complex', itself activated by binding to an anchor sequence, activates the expression of a gene after looping of double-stranded DNA; this may help to explain the mutually exclusive expression [17,18] of tandem arrays of genes. This mechanism has already been proposed [19] in the case of olfactory receptors, following the discovery of a highly conserved sequence, referred to as the 'H region', upstream of a tandem array of olfactory receptor genes. The suggestion in that case was that an activating complex is formed in the H region that interacts with one of the promoter sites activating the corresponding gene, such that the resulting DNA looping explains mutual exclusivity for the particular array of genes. When the H region was deleted, the genes in the cluster were not expressed. Similarly, we expect that when the anchor sequence of the *Dscam *gene is deleted, none of the exon 6 alternatives can be chosen. There are also other similar known cases in which such a 'locus control region' may lead to mutual exclusivity [20].

A paper with similar conclusions [21] was published while this manuscript was under review.

## Conclusion

We have described novel mechanisms that are potentially involved in the mutually exclusive exon selection in the *Drosophila Dscam *gene. Using computational methods and comparative sequence analysis, we have identified conserved intronic sequence motifs within exon cluster 6. We propose that mutual exclusive base pairing of each of these motifs with a complementary axon sequence downstream from exon 5 lead naturally to the selection of specific cluster 6 exons brought into proximity of exon 5 by the resultant 'looping' of the *Dscam *pre-mRNA. Our comparative sequence analysis also revealed a related mechanism whereby competing stem-loop structures are proposed to control a binary choice of one or the other of the pair of cluster 17 exons. These findings are important because they suggest specific experimental strategies to define the mechanisms that regulate these processes, and because the computational approach that revealed these sequence motifs can now be widely utilized to test the generality of these models for the vast majority of genes from higher eukaryotes that are also known to undergo alternative splicing. We hope that the mechanisms described here will provide valuable insights for devising methods to control splicing patterns (such as using microRNAs or proteins to obstruct part of the anchor sequence).

## Materials and methods

### Dot plots and interaction chart

The dot plots shown in Figure 4 were created using MATLAB with a window size of 11 and a minimum number of eight matches per window. We compared the reverse complement of the anchor sequence with the region upstream of each of the exons. The results were further filtered to eliminate dots that were not part of a continuous line of minimum length 4. The interaction chart shown in Additional data file 2 was created by performing Smith-Waterman alignment between the reverse complement of the anchor sequence and the region upstream of the exons, using scores 1, -1 and -2 for matches, mismatches and gaps, respectively.

### Genetic program for reconstructing anchor sequence from intron sequences

We used the score of a local (Smith-Waterman) alignment of one sequence with the reverse complement of another sequence to estimate the "strength" of a potential binding site between the two, and defined the optimization problem as finding a sequence that maximizes the sum of the scores of all such alignments. The algorithm uses as input all 47 introns of *D. melanogaster*, starting from 6.1-2 up to and including 6.47-48. It does not make any use whatsoever of the anchor sequence, which is located in a different intron, namely 5-6.1. It attempts to guess what that anchor sequence is by maximizing a score (the sum of the local alignment scores of a 'mutating' sequence with the reverse complements of all of these 47 introns). It starts from a totally random 'seed' sequence of length 30, which gradually 'mutates' in various ways, and the mutation 'survives' only if the score becomes larger. The mutation operations can be 'insertion', 'deletion', or 'substitution'. A random nucleotide is chosen as the mutation site and one of the mutation operations is chosen, each with probability 1/3. The insertion operation inserts a nucleotide and either pushes the original sequence to the right or to the left with equal probability, in which case the final nucleotide in the pushing direction is discarded so that the mutating sequence is kept at a constant length. The deletion operation deletes the nucleotide and moves the sequence either to the left or to the right with equal probability. The substitution operation changes the nucleotide to one of the others with equal probability for all nucleotides.

## Additional data files

The following additional data are included with the online version of this paper: A pdf file of the phylogenetic tree of the exons of cluster 6 (Additional data file 1); a pdf file of the interactions between the anchor sequence and the region upstream of each alternative exon of cluster six (Additional data file 2); and a zipped file of the MATLAB implementation of the genetic algorithm for automatic reconstruction of the anchor sequence (Additional data file 3).

## Supplementary Material

Additional data file 1 A pdf file of the phylogenetic tree of the exons of cluster 6 Click here for file

Additional data file 2 A pdf file of the interactions between the anchor sequence and the region upstream of each alternative exon of cluster six Click here for file

Additional data file 3 A zipped file of the MATLAB implementation of the genetic algorithm for automatic reconstruction of the anchor sequence Click here for file

## Figures and Tables

**Table 1:** Multiple exon alignment of the alternative exons of the cluster containing exon 6

*Drosophila *spp.					
*melanogaster*	*yakuba*	*ananassae*	*pseudoobscura*	*mojavensis*	*virilis*

6.1	6.1	6.1	6.1	6.1	6.1
6.2	6.2	6.2	6.2	6.2	6.2
6.3	6.3	6.3	6.3	6.3	6.3
-	-	6.4	6.4	6.4	6.4
6.4	6.4	6.5	6.5	6.5	6.5
6.5	6.5	6.6	6.6	6.6	6.6
-	-	6.7	-	-	-
6.6	6.6	6.8	6.7	6.7	6.7
6.7	6.7	6.9	6.8	6.8	6.8
6.8	-	6.10	6.9	6.9	6.9
6.9	6.8	6.11	6.10	6.10	6.10
6.10	6.9	6.12	6.11	6.11	6.11
-	-	-	-	-	6.12
6.11*	6.10*	6.13	6.12	-	6.13
6.12	6.11	6.14	6.13	-	6.14
6.13	6.12	6.15	6.14	6.12	6.15
-	-	-	-	6.13	-
6.14	-	6.16	6.15	6.14	6.16
-	-	6.17	-	6.15	6.17
6.15	6.13	6.18	6.16	6.16	6.18
6.16	6.14	6.19	6.17	6.17	6.19
6.17	6.15	6.20	6.18	6.18	6.20
6.18	6.16	6.21	6.19	6.19	6.21
6.19	-	6.22	6.20	6.20	6.22
6.20	6.17	6.23	-	-	-
-	-	-	-	6.21	6.23
-	-	-	-	6.22	6.24
-	-	-	-	6.23	6.25
6.21	6.18	6.24	6.21	6.24	6.26
-	6.19	-	-	6.25	6.27
6.22	-	6.25	6.22	6.26	6.28
6.23	6.20	-	6.23	-	-
6.24	6.21	6.26	6.24	6.27	6.29
6.25	6.22	6.27	6.25	6.28	6.30
6.26	6.23	6.28	6.26	6.29	6.31
6.27	6.24	-	6.27	6.30	6.32
6.28	6.25	6.29	6.28	6.31	6.33
6.29	-	6.30	6.29	6.32	6.34
6.30	6.26	6.31	6.30	6.33	6.35
6.31	6.27	6.32	-	6.34	6.36
6.32	6.28	6.33	6.31	6.35	6.37
-	-	-	6.32	6.36	6.38
6.33	6.29	6.34	6.33	6.37	6.39
6.34	6.30	-	-	-	-
-	-	-	-	6.38	6.40
6.35	6.31	-	6.34	6.39	6.41
6.36	6.32	6.35	6.35	-	-
6.37	6.33	6.36	6.36	-	-
-	-	-	6.37	-	-
6.38	-	-	-	6.40	6.42
6.39	6.34	6.37	6.38	6.41	6.43
6.40	6.35	6.38	6.39	6.42	6.44
6.41	6.36*	6.39	6.40	6.43	6.45
6.42	-	6.40	6.41	6.44	6.46
6.43	6.37	6.41	6.42	-	-
6.44	6.38	-	6.43	6.45	6.47
6.45	6.39	6.42	6.44	6.46	6.48
6.46	6.40	6.43	6.45	6.47	6.49
6.47	6.41	6.44	6.46	6.48	6.50
6.48	6.42*	6.45	6.47	6.49	6.51
-	-	6.46	6.48	6.50	6.52

The rows contain sets of orthologous exons, and each column refers to the species (*Drosophila melanogaster*, *D. yakuba*, *D. ananassae*, *D. pseudoobscura*, *D. mojavensis *and *D. virilis*). Alignment was achieved by clustering together exons connected by neighboring branches in the phylogenetic tree shown in Additional data file 1. Exons labeled by an asterisk (such as exon 6.11 of *D. melanogaster*, which has neither a dot plot line in Figure 4 and no observed expression level in [12]) appear as isolated leaves in the phylogenetic tree, suggesting that they are probably nonfunctional and their sequences have randomly drifted by evolution.
