# Supplementary figures and images for: Variable window binding for mutually exclusive alternative splicing
Source: Genome Biol. 2006 Jan 13;7(1):R2. doi: 10.1186/gb-2006-7-1-r2 (PMC1431710; doi:10.1186/gb-2006-7-1-r2)

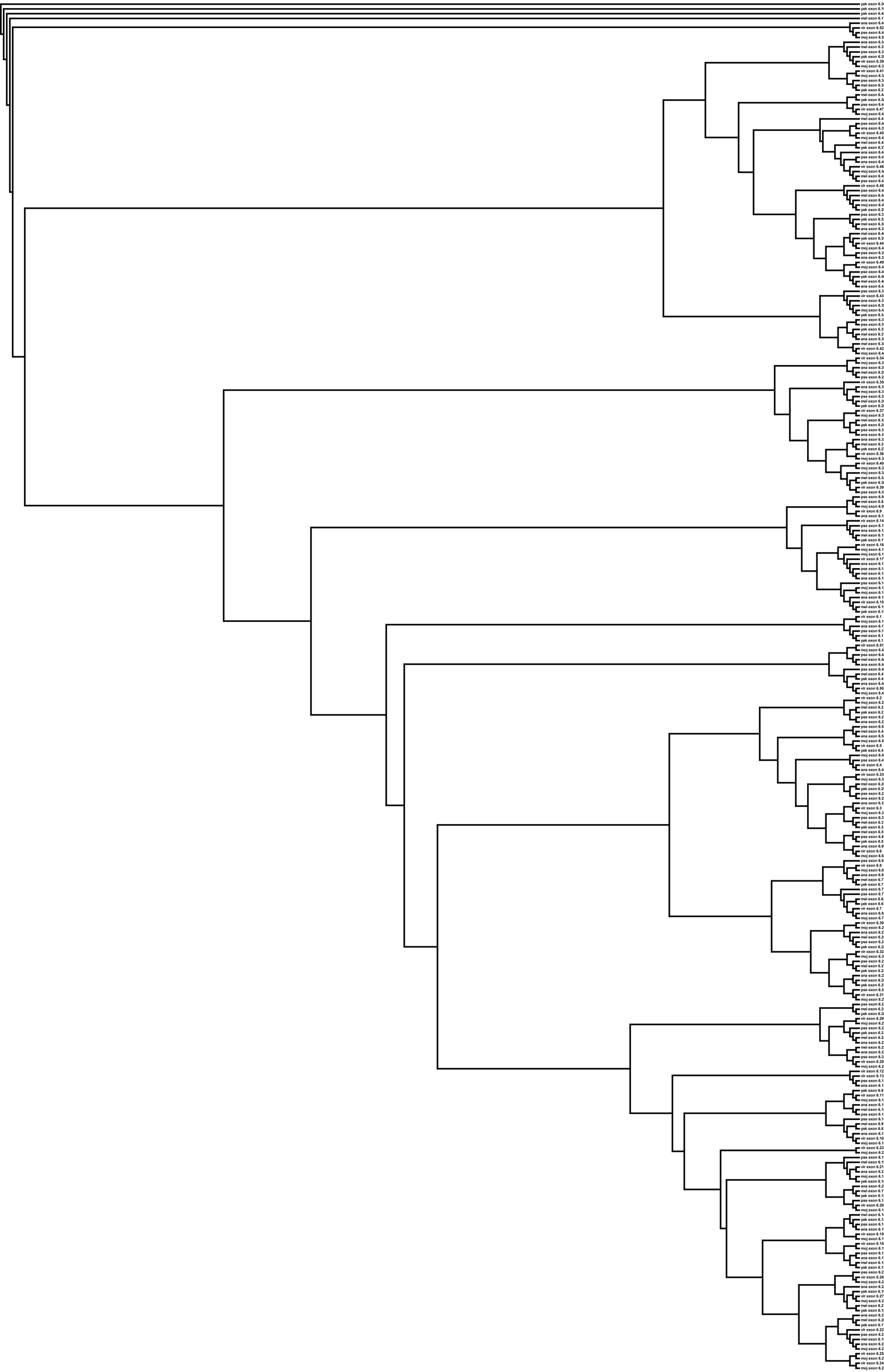

Supplement: Additional data file 1 — A pdf file of the phylogenetic tree of the exons of cluster 6 [file gb-2006-7-1-r2-S1.pdf]
