# Supplementary material for: Variable window binding for mutually exclusive alternative splicing
Source: Genome Biol. 2006 Jan 13;7(1):R2. doi: 10.1186/gb-2006-7-1-r2 (PMC1431710; doi:10.1186/gb-2006-7-1-r2)
Supplement: Additional data file 2 — A pdf file of the interactions between the anchor sequence and the region upstream of each alternative exon of cluster six [file gb-2006-7-1-r2-S2.pdf]

EXON NUMBER	ALIGNED SEQUENCE	ALIGNMENT SCORE
1	TTGTCGAGTACCCTATC-CCAACATTCAGGCAGTTTTCAATTT<br>TATTCTCCTTTGAACTGCGCTCAAGGTAAGTCACTTCACATCCACTTCCATATCTTCACCAATT-GGCATTTTACTATGTAAGGCTATTAACTTTGAACTATATTTCGCTTCATTCACAG	10
2	TTGTCGAGTACCCTATCCCAACATTCAGGCAGTTTTCAATTT<br>TTTTAGGTAGGACTAATATCCATCCAG-ACCC-AGACCAACTTGC-GCCAGTTTCCAATTGAGATTGCTCGCCTTAGTTGTTATTAGCATAATCCAGCATAATTAGCTTCCTCTTTCAAG	15
3	TTGTCGAGTACCCTATCCCAACATTCAGGCAGTTTTCAATTT<br>TGTCCAGCTCAATCATATCCAGTGCCAGTCTTTAGGTAAGACATACGCCCATCTGCCTATCCCAACTGAAGGTGTTCCTTTCGAGAATTAAATATTGGGTTCCCCTCTTCGAAATCCCAG	11
4	TTGTCGAGTACCCTATCCCAACATTCAGGCAGTTTTCAATTT<br>AACTCTTCTCTGCCCGGCTCAGGCTTATCCGGTGCCCTTCTTTAGGTAAACCCAA-CCCAACACTTAGGCTTATATATGTAGATTACGTTTCCGGTCTATTAATATGCCGAATTCAACAG	12
5	TTGTCGAGTACCCTAT-CCCAACATTCAGGCAGTTTTCAATTT<br>TGTCCGGCTCAAGCCTATCCAGTGCCCTTCTTTAGGTAACAATCCTCCTAATTTTATGCCCAAC-TTCAGGCAGGGTCTAGAATCTAAAAGGTTAATTTTTATGTGAAACCTCCTTTAAG	14
6	TTGTCGAGTACCCTATCCCAACATTCAGGCAGTTTTCAATTT<br>GTGCCGGTGTATAGGTAAAATCAGCTCTTTTATTTCAGTC-AGT-CCCT-TCCCATC-T-CAGGCACAAAATGATCTTGTAGTGGTTTGAACTAGAATCCCTAACTATTCTTCCTTATAG	12
7	TTGTCGAGTACCCTATCCCAACATTCAGGCAGTTTTCAATTT<br>GCGTATCCACAGCCTTTCTTTAGGTAAAAGCTCCACAAGAATATCCCTGACTTTCACTTC-GGCAGTTTACAATTGAGTTAGGAATCTTGTACGAAAATGAAACGAATTTAACCTTGCAG	13
8	TTGTCGAGTACCCTA-TCCCAACATTCAGGCAGTTTTCAATTT<br>CTATGTCCAGCTCAGGCATATCCACAGCCAATATCTAGGTAACTTTCAACTCCTAGGCCCAACATTCAAGAAAAAGATACCTGCTTTCTACATGGGGATAGCTAATTTTCGATTTTGTAG	12
9	TTGTCGAGTACCCTATCCCAACATTCAGGCAGTTTTCAATTT<br>GTCCGGTACAATCGTATCCCATGCCTGTTTTCAAGTAAGATTCAGTGAGGTTTTACTTTAAATTAAAATCCAAACACATTCAGTCAGTTG-CAATAAGGGAAAAAATCTTTAAATTTTAG	13
10	TTGTCGAGTACCCTATCCCAACATTCAGGCAGTTTTCAATTT<br>CTCCTGTGCATGGCTCAGTCGTATCCTACCCCGTCCTTCAGGTGGGTTTCCCTTTCCCAACATCATCATCAGACAGTTTTCCTTGCTTTTCCGTGACTCTCCTTGATGTTTTGCCCGTAG	15
11	TTGTCGAGTACCCTATCCCAACATTCAGGCA-GTTTTCAATTT<br>TTCCCAGGGAATCGGAATCGCTCTACTGTGTCCGGCGCAGTCATATCCGATTCCAGCCCACAGGTTTGCATGCCATCAATTGGGAACATTTATAATTTACAGGGCGGTATTCGAATTCAG	8
12	TTGTCGAGTACCCTATCCCAACATTCAGGCAGTTTTCAATTT<br>CATCCCAGACATATCCCCTTCCCGCTTGTAGGTAGGTAGTCTTTACTTGCTCAACTTCCAACATTTGGGCAGATTTCCTGGGTATATATCCCAGTCCTTAAAGTCTTATGTTTCGCTCAG	14
13	TTGTCGAGTACCCTATCCC-A-A-C-ATTCAGGCAGTTTTCAATTT<br>GCCCTGGTCAGGCATTTCCAGTACCCATTAACAGGTAATACGTAGCCCATCCCAACACCAATTCAGACTGTTTTTTTTTATTGGGACATCCTGAGGATTTCAATATACTTCATATTCCAG	13
14	TTGTCGAGT-ACCCTATCCCAACATTCAGGCAGTTTTCAATTT<br>TCCCGGTCAGGCATATCCAGTTCCGGTCGTTAGGTGGGTGTCGTTTCATTCTATCCCAGCATTCCAGATAGATGATTTTTGAGAAATATTTCCCCTAATAGTATTGTTTCAAACAATCAG	13
15	TTGTCGAGTACCCTATCCCAACATTCAGGCAGTTTTCAATTT<br>CTCCATGCAATGTCCTGGGCAGGCATACCCGGTGCCAATTGTTAGGTTAGTCTCGA-TGCCCTCCACCAACATTCACTTTTGTATGCCGAAGAATTAACTTGAAATTATCTTAAACCCAG	13
16	TTGTCGAGTACCCTATCCCAACATTCAGGCAGTTTTCAATTT<br>CCTCCAAGGCTCTCATCTGTCCAGCTCAGTCGTATCCAATGCCGTTCTTTAGGTAAATACCCCTGTCC-AACATTCAGGTATTTCTTAGCATGGCCTACTAAGGAGTCAATTTCTCTTAG	15
17	TTGTCGAGTACCCTATCCCAACATTCAGGCAGTTTTCAATTT<br>AGTCTTATCCAGTGCCTGCCTTCCGGTGAGTACCAGCTGTC-AGGACT-TGTCCCAGCACTTAGGCAGTAAATCGAAATCGGCAGATGCCATGACTAAGCTCCCCCCTAAATCTTTGTAG	14
18	TTGTCGAGTACCCTATCCCAACATTCAGGCAGTTTTCAATTT<br>GTCCAGCTCAATCCTTTCCCATGCCAAGCGCTCGGTAAGCTTTCAGGCAGTTTTCTTAAAAAGATCTTCTTAAGCTCATATGTTTTTGGAGATTGGATTTAGCACTTGTACTATTTGCAG	14
19	TTGTCGAGTACCCTATCCCAACATTCAGGCAGTTTTCAATTT<br>CCATGCCAGTTTATAGGTAGGAATATCTCTTCTCTGTCTTCGTCAGGCAGTTTTCAAAGTTTCCTTTAGCTGATAGGTGTGCTATAGTACAATACATGCAGTTCTTACTGAACCTTTCAG	15
20	GTCGAGTACCCTATCCCAACATTCAGGCAGTTTTCAATTT<br>CAAGGATTTCCTATGCCGGCATTTAGGTAATCACATTGCTGAGTACAGTCCACAGGCCACGGTTTTCATGAGATTGGGATCATTAGTTGTATCTATACCTCCGATATACCTTTCTTACAG	10
21	TTGTCGAGTACCCTATCCCA-ACATTCAGGCAGTTTTCAATTT<br>CTCAAGGTTATCCAATGCCAGCGTTTAGGTAAAAACATTGTTGAGTACA--ATCCTACAGAT-C-GG--GTTTTCCATTTACATAGAATGTTTAGAAGCAATATAAGAATATAATTTCAG	12
22	TTGTCGAGTACCCTATCCCAACATTCAGGCAGTTTTCAATTT<br>GCCCAGCTCAATCGTATCCAATCCCAGCTTTTAGGTAAACACTTAAGATTTAAATTGTCCAGTACGTGAAACCATTCTGGCAGATTTTTTGAATCAAAACTAAGATAAAACCTCCCACAG	12
23	TTGTCGAGTACCCT-ATCCCAACATTCAGGCAGTTTTCAATTT<br>CAATGTCCAGCTCAGGCCTTTCCGGTTCCGGTTTATAGGTAATGTCGAGTGCCCTGGTTGCCTTATTCATGTAGTTTTCATGGTCTATGATGGTCTTACGTAATTCGGCACTGTTTCCAG	17
24	TTGTCGAGTACCCTATCCCA-ACATT-CAGGCAGTTTTCAATTT<br>GCACAGTCCTTTCCAGTGCCCGTTTATAGGTTGGTCATTGTCGAGAGCTCTTTACATCCAATACTTTCCAGGCAGTCGAGGCAGTTCATTCCCATCATAAATCTCATCTTAAATTTGTAG	11
25	TTGTCGAGTACCCTATCCCAACATTCAGGCAGTTTTCAATTT<br>CCCCTTCCGCTGCAAAACTTAGTTGAATGACAGGTGGCTTCCAATTGTGTCGAGTTCCTTGGGCAGTTTTCAATCTCAGCACGGGTTTTCGGTTCTCATCCCCACTTCCATTGCTTCCAG	13
26	TTGTCGAGTACCCTATCCCAACATTCAGGCAGTTTTCAATTT<br>TATTTTGTCCCGCCCAATCCTATCCGGTTCCTGCATTCAGGTGAGCCCCATCCATTTCCACTCAGGCAGTTTTCACTAGACTTCGGTTAGCTGGCACTTTCAATATTCATTATCTTTCAG	15
27	TTGTCGAGTACCCTATCCCAA---CATTCAGGCAGTTTTCAATTT<br>CTTTTGTCCAGCTCAATCGTATCCGGTACCATCGTTTAGGTAATCCCAAGTTCGTTCAGGCAGACTTCAAACTGACTTTTAAATTCTCAAATGTTTTACTAAATGGGCAATCTCTTTTAG	13
28	TTGTCGAGTACCCTATCCC-A-ACA-TTCAGGCAGTTTTCAATTT<br>CTTCACCCTCTTTTGCCCTGGTCAAGCCTATCCAGTACCCTTCTTTAGGTAAATCCCTACGCATTTTAGGCAGTTTTCCGGTTTACTTAGTTCTAGTGTTGTATACCCTCTGTTTTATAG	13
29	TTGTCGAGTACCCTATCCCAACATTCAGGCAGTTTTCAATTT<br>TGTCCAGCCCAGGCATATCCGGTACCATTCTTCAGGTGATTCTGCTCAGACATTCAGGGAGTTTTAAGTTATGGCTGTCTTGGAGTGAATCGTTATGGAATATTATGATTTATGTTTCAG	15
30	TTGTCGAGTACCCTATCCCAACATTCAGGCAGTTTTCAATTT<br>TCTATAATGTGTCCTGCGCAGGGTTTTCCCGTGCCAGCTTTTAGGTGAGGTCCAACATTCGGGGAGTTTTCAATAAACAATAGTTTTCCTTTGGTAAAATGGTGTTATTAACCTTTTTAG	19
31	TTGTCGAGTACCCTATCCCAACATTCAGGCAGTTTTCAATTT<br>CTACCCGGTGCCTGTGTTTAGGTAATCAAAGCGGAAAGATTGGGAATCAGTGTCAATATTCAGGCAGTTGTATGGAGTTGTTAGAGCCTTCTATAGTCGCAAATTGAAATCTTTTTGCAG	14
32	TTGTCGAGTACCCTATCCCAACATTCAGGCAGTTTTCAATTT<br>TCGGTTTTCTTCCAAAATAGGAAAATACATTTTTTGCTTCTTATGATTCCCA-CATTCAGACAGTTCGCGGCACTTACTCTCCTTAACTTTTGTAACTTTGTAACTTTTGCCTTTCGTAG	14
33	TTGTCGAGTACCCTATCCCAACATTCAGGC--AGTTTTCAATTT<br>GTTCTACAGATTCTGTAGGGGCCTTGAACCAACAT-CAGGCTTGGTTTTCGATTTCCTTTTACATAGAAACACTATAAAACTATAACAAGTTAAATCCATATACATATGTACGTATGTAG	14
34	TTGTCGAGTACCCTATCCCAACATTCAGGCAGTTTTCAATTT<br>TTTCTAGATTCCCCCTCAGAGATGAAGTAACAAATAAATAGGGTACTGTTCAGCCCTTAGACCAACAT-CAGGCTGGGATATCGATCCATAAGAAATACTTCACCATAATCTCTTCACAG	11
35	TTGTCGAGTACCCTATCCCA-ACATTCAGGCAGTTTTCAATTT<br>ACTTGTTTTATTGCGCTTACCAAATACCACGTAGAAAGATCCCTTATAGCAAGCTTACTTCCTATCCCATACATTCGTGCAATGTCCGCGATAGATTCTAATACTGCCATCATCCCACAG	14
36	TTGTCGAGTACCCTATCCCAACATTCAGGCAGTTTTCAATTT<br>GTGAAAGTTTGTTGCTTTGCATTAAATGGAAAGTATTTGATGACTTTCGTCGACCCCCAAGCACATTCAGTCAGTTTATTGTTTGCGTTTAGCTTAATTAATGGGTGACTTAATTCACAG	13
37	TTGTCGAGTACCCTATCCCAACATTCAGGCAGTTTTCAATTT<br>CCAAGCCTTTCCAGTGCCCATCATTAGGTAAGCCCCACATTCAGGCAGTTAGTAAGTTACTAGTGGTATGATTTTTGGAACACAACTTAATCAGAATGCCAACTTATTAAAATTTCCCAG	16
38	TTGTCGAGTACCCTATCCCAACATTCAG-GCAGTTTTCAATTT<br>TTTTAGGTATATACAATTTTTTGGTTGAAGAAAGTAAGTCAGGCCCGAAATTTCAGCACTCAGAGCAGTTAGATGTGAATTGTGGTCTTTAGAGTTAAAATTGTACGTGTAACTTTGCAG	10
39	TTGTCGAGTACCCTATCCCAACATTCAGGCAGTTTTCAATTT<br>ATTCAAATATACTCCACTTCTTTATGTTAAAATGGGTCGAGCTGTTGATAAACCCAATTTTCAGTCAGTTTTCATTTAAGTGCATTTTCCTGCCTTAATAAATGTATTTCAACTCTTTAG	17
40	GTCGAGTACCCTATCCCAACATTCAGGCAGTTTTCAATTT<br>CAATCCTATCCTGTACCAATATTTAAGTAA--TTCAATTCAATCCAACGCTGTTCAACTGGTTCTGTTAGGGTTCAAATAGAAATTAATGCCAAAGACATATTACATTTATCTATTATAG	11
41	TTGTCGAGTACCCTATCCCAACATTCAGGCAGTTTTCAATTT<br>AGCGGTCGATTAAGGCCAGCTTTGCTCTTTTGTGCCAGGCTCAGGCGTTCCCCGTTCCCATCATT-AGGTAAGTTTCAGCAAACAGGCTTCTAGGTTAATCCTCTTTAAAAACATTGCAG	13
42	TTGTCGAGTACCCTATCCCAACATTCAGGCAGTTTTCAATTT<br>CGATGCTTTGCCAGGCGCAGGCTTTTCCACTGCCCATAATGAGGTTAGCCTTGATCCCGTCGTAATCAGGCAGTTTTCATAGAGATTAGTCACTTTTAAATTGGTTAATATTATTTGCAG	14
43	TTGTCGAGTACCCTATCCCAACATTCAGGCAGTTTTCAATTT<br>TTGTCAGGCTCAGGCATATCCAGTTCCCATTATGAGGTTAGATATTATAACTATTTAGCAT-CAGGCAGTTTTCCCATATACAAATCCCTTGAACTAAAGTTCCAAGTCATTGTTTTCAG	13
44	TTGTCGAGTACCCTATCCC-AA-C-ATTCAGGCAGTTTTCAATTT<br>CTCAGGCCTATCCAGTTCCCGCTATGAGGTACTACATGACCTAAACCCAAACCTATTCAAGCAGTTAATTAAGCGACCCTCAAAACAATCTTAAGTTTCCAAATTTATTCGAACTTTCAG	11
45	TTGTCGAGTACCCTATCCCAACATTCAGGCAGTTTTCAATTT<br>CTACTCGGCTCGAGTTCGAGTGATATTGTGCTACTTTGCCAGGCTCAGGCTTTTCCAGTTCCCTATACACGGTAATTGCAGTTCAGGCAGCTTAGAAGGCTTTTCTTTCTTGTTTACTAG	10
46	GAGTACCCTATCCCAACATTCAGGCAGTTTTCAATTT<br>TCGCGTTACTCTGCCAGGCGCAGGC-GTATCCAGTGCCATTGATAAGGTAGCCAATAAGTAGACTTAAGACTACAACATCAATAAGTCCCATAAGAAGCATATTGTTATTACTTTTGTAG	6
47	TTGTCGAGTACCCTATCCCAACATTCAGGCAGTTTTCAATTT<br>ACTAGTTTCGCCCTTTTGTGCCAGGCGCAGGCTTTTCCAGTACCCATTATCAGGTTAGTCCAACATTCGGGCA-TATCCAATTAGACAGAGGGGCCATCTTTACGTAAGTGCTGATCCAG	15
48	TTGTCGAGTACCCTATCCCAACATTCAGGCAGTTTTCAATTT<br>CTTGGGACAATCGTTTCCCACACCCGCATTTAGGTAATGAAAGCTATTTATCAGAGCCAACATTCAGGCAGCAAATAGCATTTCAAATAGGGATCTTACAGAATTTTGCTTTGATTTCAG	15
